# Writers, Readers, and Erasers of Histone Ubiquitylation in DNA Double-Strand Break Repair

**DOI:** 10.3389/fgene.2016.00122

**Published:** 2016-06-28

**Authors:** Godelieve Smeenk, Niels Mailand

**Affiliations:** Ubiquitin Signaling Group, Protein Signaling Program, Faculty of Health and Medical Sciences, The Novo Nordisk Foundation Center for Protein Research, University of CopenhagenCopenhagen, Denmark

**Keywords:** DNA damage response, ubiquitin, DNA double-strand breaks, chromatin, DNA repair, histones

## Abstract

DNA double-strand breaks (DSBs) are highly cytotoxic DNA lesions, whose faulty repair may alter the content and organization of cellular genomes. To counteract this threat, numerous signaling and repair proteins are recruited hierarchically to the chromatin areas surrounding DSBs to facilitate accurate lesion repair and restoration of genome integrity. In vertebrate cells, ubiquitin-dependent modifications of histones adjacent to DSBs by RNF8, RNF168, and other ubiquitin ligases have a key role in promoting the assembly of repair protein complexes, serving as direct recruitment platforms for a range of genome caretaker proteins and their associated factors. These DNA damage-induced chromatin ubiquitylation marks provide an essential component of a histone code for DSB repair that is controlled by multifaceted regulatory circuits, underscoring its importance for genome stability maintenance. In this review, we provide a comprehensive account of how DSB-induced histone ubiquitylation is sensed, decoded and modulated by an elaborate array of repair factors and regulators. We discuss how these mechanisms impact DSB repair pathway choice and functionality for optimal protection of genome integrity, as well as cell and organismal fitness.

## Introduction

Conserving the integrity of DNA and the information stored within its sequence is critical for the viability and fitness of any living cell and organism. DNA is continuously subjected to genotoxic insults inflicted by endogenous as well as exogenous sources (Barnes and Lindahl, 2004; Jackson and Bartek, 2009). Among the resulting spectrum of DNA lesions, one of the most cytotoxic types is the DNA double-strand break (DSB; Wyman and Kanaar, 2006). If left unrepaired or repaired incorrectly, such breaks can give rise to mutations or chromosomal rearrangements, which may lead to cell death or cancer development. In parallel with these stochastic DSBs, programmed DSBs play a central role in various biological processes in both uni- and multicellular organisms. These intentional DSBs are generated to facilitate the exchange of genetic information between homologous chromosomes during meiosis in diploid and polyploid organisms, as well as in processes such as mating type switching in haploid yeast (De Massy, 2013). In addition, programmed DSBs are instrumental for the genetic exchanges required for T-cell receptor rearrangement and V(D)J- and class-switch recombination (CSR) during lymphocyte maturation (Alt et al., 2013). The potentially malignant consequences of improperly processed DSBs on human physiology are illustrated by the fact that many leukemias result from chromosomal translocations caused by faulty rejoining of programmed breaks at the immunoglobulin locus in B cells (Alt et al., 2013).

Two major pathways are employed by eukaryotic cells for the repair of DSBs. Non-homologous end-joining (NHEJ) is active throughout interphase and promotes rapid religation of broken DNA ends that do not require extensive end-processing, but is an error-prone process (Lieber, 2010). Homologous recombination (HR) mainly functions during S and G2 phases, when a newly replicated sister chromatid is available as a template for error-free repair (Heyer et al., 2010). This pathway is used for the repair of more complex DSBs and occurs with slower kinetics than NHEJ. HR is initiated by resection of the broken DNA ends by the Mre11-Nbs1-Rad50 (MRN) complex, CtIP, and EXO1 and BLM-DNA2 exonucleases. The choice of pathway for repair of individual DSBs is influenced by several parameters, including cell cycle status, the complexity of the break and whether it resides in euchromatic or heterochromatic regions of the genome (Shibata et al., 2011; Chapman et al., 2012b; Ceccaldi et al., 2016).

To counteract the threat posed by potentially deleterious DNA lesions, cells have evolved a complex network of interwoven, protective pathways that are collectively referred to as the DNA damage response (DDR; Jackson and Bartek, 2009; Ciccia and Elledge, 2010). DSBs are particularly potent triggers of the DDR, stimulating DNA repair pathways, transient arrest of the cell cycle, and transcriptional reprogramming. A striking feature of the cellular response to DSBs is the massive accumulation of DDR factors directly at the sites of DNA damage. The resulting structures, often referred to as Ionizing Radiation Induced Foci (IRIF), can be readily visualized by immunofluorescence microscopy (Bekker-Jensen and Mailand, 2010; Lukas et al., 2011). To a large extent, this rapid accumulation of proteins at DSBs relies on DNA damage-induced posttranslational modifications (PTMs) of histones and other chromatin-associated proteins that are in turn recognized by specific effector proteins (Lukas et al., 2011; Polo and Jackson, 2011). The functional consequences of this DSB recruitment programme range from chromatin relaxation to protection of the broken ends and assembly of repair protein complexes (Bekker-Jensen and Mailand, 2010; Lukas et al., 2011). Inherited mutations in genes encoding DSB signaling factors are associated with cancer and other genetic instability syndromes, illustrating their pivotal importance for DDR functionality and genome maintenance. For example, biallelic mutations in the *RNF168* gene, which encodes a ubiquitin ligase that catalyzes histone H2A ubiquitylation near DSBs to attract downstream repair factors, is the underlying cause of the ataxia-telangiectasia-like RIDDLE syndrome (Stewart et al., 2009). Patients with this rare disease present with symptoms typical of genomic instability syndromes, including radiosensitivity, immunodeficiency, and neurodegeneration (Stewart et al., 2007; Devgan et al., 2011).

A large body of work has given rise to a model in which DSB formation is accompanied by the propagation of a DNA damage-induced histone code that is written, read and ultimately erased by an elaborate network of effector proteins and regulators. Central to this process is the ubiquitylation of histones in the vicinity of DSBs by the two E3 ubiquitin ligases RNF8 and RNF168, coupling DSB detection to efficient repair of the lesions. In this review, we summarize and discuss how RNF8- and RNF168-mediated chromatin ubiquitylation orchestrates DSB signaling and repair mechanisms in mammalian cells, and how the DSB-associated histone ubiquitylation marks generated by these E3s are subsequently interpreted and turned over during the course of DNA repair to protect genome stability.

## Writers of DSB-associated histone ubiquitylation

The formation of DSBs sets in motion a cascade of signaling events that collectively facilitates faithful repair of the lesions. DSBs trigger rapid activation of the ATM kinase in a process that involves its acetylation by TIP60 (KAT5), induced by chromatin alterations (Sun et al., 2007, 2009; Kaidi and Jackson, 2013). A key target of activated ATM is the histone H2A variant H2AX, which contains a unique ATM phosphorylation site in its C-terminal tail (Rogakou et al., 1998). The product of this phosphorylation event, known as γ-H2AX, provides a binding site for the MDC1 protein via its tandem BRCT domain, a phosphopeptide-binding module found in a range of DDR proteins (Stucki et al., 2005; Mermershtain and Glover, 2013). MDC1 is a scaffold protein that recruits a number of factors to DNA damage sites. Among these is the E3 ubiquitin ligase RNF8, which initiates a dynamic ubiquitin-dependent DSB signaling response that culminates in the generation of specific ubiquitin marks on H2A-type histones near the breaks, laid down by another E3 ligase, RNF168 (Huen et al., 2007; Kolas et al., 2007; Mailand et al., 2007; Doil et al., 2009; Pinato et al., 2009; Stewart et al., 2009; Thorslund et al., 2015). These ubiquitin modifications at damaged chromatin serve as recruitment platforms for a range of important DSB repair factors. The DSB signaling response thus undergoes a switch from being extensively driven by phosphorylation, targeting H2AX and associated factors, to relying also on a wave of ubiquitylation events mediated by RNF8, RNF168 and other ubiquitin ligases.

**Figure 1:**
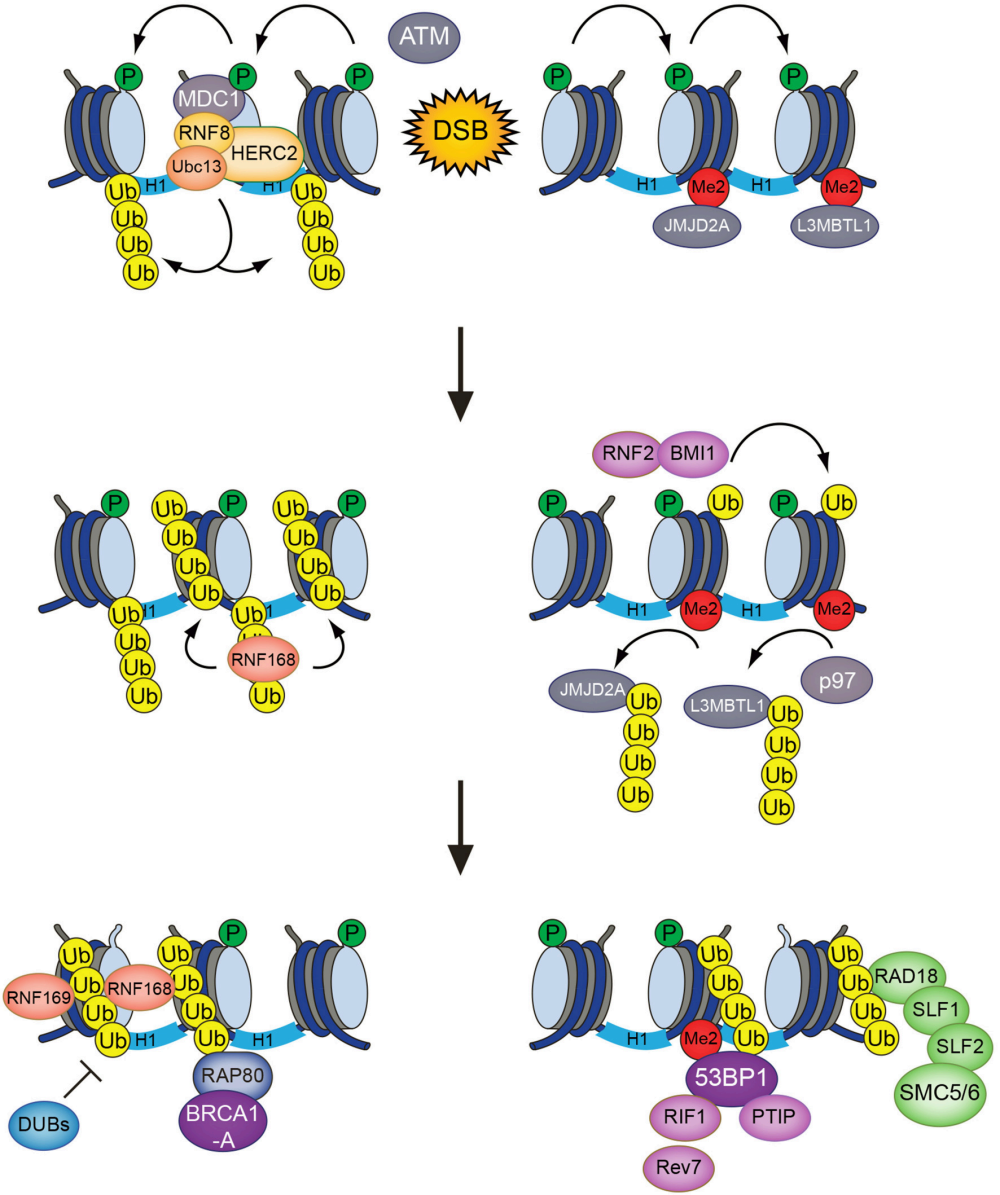
**The RNF8/RNF168-mediated histone ubiquitylation pathway**. DSB-associated histone ubiquitylation requires the sequential action of the RNF8 and RNF168 ubiquitin ligases. RNF8 is targeted to DSB sites through interaction with phosphorylated MDC1, promoting Ubc13-dependent K63-linked polyubiquitylation of H1-type linker histones. This serves as a recruitment signal for RNF168, which catalyzes and propagates ubiquitylation of H2A-type histones at K13/K15 to an expanded chromatin area surrounding the break sites. These modifications provide loading platforms for a range of important components of DSB repair pathways, including 53BP1, BRCA1 (via RAP80), and the SMC5/6 complex (via RAD18-SLF1-SLF2), which have key roles in promoting DSB repair efficiency, fidelity, and pathway choice.

RNF8 is recruited to sites of DNA damage via its FHA domain, which recognizes ATM phosphorylation sites in MDC1 (Huen et al., 2007; Kolas et al., 2007; Mailand et al., 2007; Figure 1). While it has long been clear that RNF8 collaborates with the E2 ubiquitin-conjugating enzyme Ubc13 to deposit K63-linked ubiquitin chains at DSB sites (Huen et al., 2007; Kolas et al., 2007; Mailand et al., 2007), the identity of its chromatin-bound substrate(s) has been more puzzling. Initially, RNF8 and RNF168 were thought to share H2A-type histones as substrates. Recently, however, it was shown that RNF8 is inert toward ubiquitylation of nucleosomal H2A and mainly promotes K63-linked polyubiquitylation of H1 linker histones but not core histones at DSB sites (Mattiroli et al., 2012; Thorslund et al., 2015). This ubiquitylation event serves as a recruitment signal for RNF168, which in turn ubiquitylates H2A-type histones at K13/K15 (Gatti et al., 2012; Mattiroli et al., 2012; Fradet-Turcotte et al., 2013). RNF168 is recruited to DSBs by recognizing ubiquitylated histone H1 via its UDM1 region, composed of two ubiquitin-binding motifs (MIU1 and UMI) and a flanking target recognition motif (LRM1; Panier et al., 2012; Thorslund et al., 2015). At the break sites, RNF168 potently catalyzes ubiquitylation of H2A-type histones at the N-terminal K13/15 residues. The ability of RNF168 to also bind ubiquitylated H2A via its C-terminal UDM2 motif, composed of MIU2 and LRM2, enables it to efficiently propagate this modification to the surrounding chromatin areas once recruited (Gatti et al., 2012; Mattiroli et al., 2012; Panier et al., 2012). The acidic patch on the nucleosomal surface, composed of residues in H2A and H2B, plays an important role in promoting N-terminal H2A ubiquitylation by RNF168. Changing essential residues in this patch impairs the binding of RNF168 and subsequent H2A-K13/K15 ubiquitylation (Leung et al., 2014; Mattiroli et al., 2014). As it is becoming apparent that RNF8 and RNF168 target different histone substrates at DSB sites, an important question concerns the nature of RNF168-generated ubiquitylations on H2A-K13/K15. Early studies showed that RNF168 deposits K63 linked ubiquitin chains on H2A-type histones, in part because it was found to interact with Ubc13, which exclusively catalyzes K63-linked polyubiquitylation in conjunction with its partner proteins Uev1 or Mms2 (Hofmann and Pickart, 1999; Doil et al., 2009; Stewart et al., 2009). However, a recent *in vitro* study suggested that, at least when overexpressed, RNF168 promotes K27-linked ubiquitylation of H2A(X), and that these atypical ubiquitin chains have an important role in promoting DSB signaling (Gatti et al., 2015). The nature of the cognate E2 enzyme(s) that catalyze K27-linked ubiquitylation in conjunction with RNF168 remains to be established. Another study showed that RNF168 forms ubiquitin chains together with UbcH5c, but not Ubc13, suggesting that RNF168 may not primarily assemble K63-linked polyubiquitin chains at damaged chromatin (Mattiroli et al., 2012). Indeed, RNF168 is capable of promoting recruitment of repair factors to DSB sites even in cells lacking Ubc13 (Thorslund et al., 2015). Moreover, it was shown that 53BP1, a direct reader of RNF168-generated ubiquitylation, binds to monoubiquitylated H2A-K15 (Fradet-Turcotte et al., 2013), suggesting that at least in some cases, RNF168 can drive protein recruitment to DSB sites without assembling polyubiquitin chains. Thus, it is possible that RNF168 has several substrates at damaged chromatin that are differentially ubiquitylated, or that RNF168 catalyzes different types of ubiquitin modifications on H2A. It is also conceivable that RNF168 may, at least in part, amplify K63-linked ubiquitylation at DSB sites indirectly by promoting the robust accumulation of other E3 ubiquitin ligases.

Although RNF8 and RNF168 are pivotal ubiquitin ligases in DSB signaling, additional layers of histone ubiquitylation and E3s are also involved, highlighting the complexity of chromatin ubiquitylation at DNA damage sites. RNF8 interacts with an auxiliary E3 ligase, HERC2, via its FHA domain that recognizes a specific C-terminal ATM phosphorylation site in this protein (Bekker-Jensen et al., 2010). The association with HERC2 appears to facilitate preferential binding of RNF8 to Ubc13 among its cognate E2 ubiquitin-conjugating enzyme partners. Due to its sheer size, however, the precise function(s) of the 530-kDa HERC2 protein and its intrinsic ubiquitin ligase activity in the DSB response has not yet been conclusively established.

Canonical monoubiquitylation of H2A on K119 is a highly abundant chromatin modification, generated by the E3 ligase heterodimer BMI1 (RING1a) and RNF2 (RING1b), which forms the stable core of the Polycomb Repressive Complex 1 (PRC1; Wang et al., 2004). Monoubiquitylation of H2AK119 plays a fundamental role in transcriptional silencing of genes, but BMI1 and RNF2 also accumulate at DSB sites, suggesting that gene silencing takes place at DSBs (Chou et al., 2010; Facchino et al., 2010; Ismail et al., 2010; Ginjala et al., 2011; Pan et al., 2011; Wu et al., 2011). Indeed, a reporter system visualizing transcription at DSB sites showed that transcription is silenced locally when DSBs are induced (Shanbhag et al., 2010). Interestingly, both inhibition of RNA polymerase I and II activity at DSB sites is dependent on ATM activation and RNF2/BMI-dependent ubiquitylation of H2AK119 (Kruhlak et al., 2007; Shanbhag et al., 2010; Kakarougkas et al., 2014; Larsen et al., 2014; Harding et al., 2015; Ui et al., 2015). Whether ubiquitylation of H2AK119 and H2AK13/K15 at DSB sites initiate separate pathways that facilitate transcriptional silencing and DSB signaling, respectively, or whether the PRC1 complex also has a more direct role in the latter process remains an important outstanding question.

Similar to H2AK119, H2B is monoubiquitylated at K120 in vertebrates (Thorne et al., 1987). However, whereas H2A K119 ubiquitylation induces transcriptional repression, ubiquitylation of H2B is associated with active transcription. The monoubiquitylation of H2B is catalyzed by the E2 enzyme RAD6 in conjunction with the E3 heterodimer RNF20-RNF40 (Kim et al., 2009). The mechanism of H2B ubiquitylation in transcriptional elongation has been studied extensively in yeast (Henry et al., 2003; Wood et al., 2003), and a similar mode of action has been found in human cells. When RNA Polymerase II encounters a nucleosome, the PAF1C transcription complex is recruited, which in turn binds RNF20-RNF40-RAD6 to ubiquitylate H2B (Zhu et al., 2005; Kim et al., 2009). The histone chaperone FACT, which also binds to RAD6 and ubiquitylated H2B, in turn shuttles away the other H2A-H2B dimer to facilitate nucleosome reorganization and chromatin relaxation (Pavri et al., 2006; Hondele et al., 2013). H2B ubiquitylation is a prerequisite for methylation of H3K4 and H3K79, which are associated with active transcription, potentially explaining, at least in part, the role of H2B ubiquitylation in this process (Briggs et al., 2002; Ng et al., 2002; Sun and Allis, 2002). Interestingly, RNF20 and RNF40 are also recruited to DSB sites, suggesting that local monoubiquitylation of H2BK120 takes place near DSBs (Moyal et al., 2011; Nakamura et al., 2011). In this context, H2B ubiquitylation might trigger conformational changes leading to H3K4 methylation or the *de novo* formation of this mark, generating a recruitment platform for the chromatin remodeler SNF2h (Nakamura et al., 2011). This in turn promotes chromatin relaxation and facilitates efficient recruitment of factors involved in HR (Moyal et al., 2011; Nakamura et al., 2011; Oliveira et al., 2014). The FACT complex was also found to be required for SNF2h accumulation at DSB sites, suggesting a mode of action similar to that at transcriptional sites (Oliveira et al., 2014). The opposing effects of H2AK119 and H2BK120 monoubiquitylation on transcription could be explained by the finding that H2BK120 ubiquitylation sterically disrupts chromatin compaction, while H2AK119 ubiquitylation, which lies at the other side of the nucleosomal surface, does not have the same effect (Jason et al., 2001; Fierz et al., 2011). Instead, H2AK119 ubiquitylation could act as a binding platform for proteins that facilitate transcriptional repression or DSB signaling, similar to H2AK13/K15 ubiquitylation.

Finally, a role of the E3 ligase BBAP in conjunction with its binding partner BAL1 (PARP9) in catalyzing monoubiquitylation of H4K91 has been reported to be a PARP-dependent mechanism acting in parallel with the RNF8/RNF168 pathway to enhance the accrual of DSB repair factors including 53BP1 and BRCA1 (Yan et al., 2009, 2013). However, although BBAP and BAL1 accumulate at DSB sites, it is not yet known whether H4K91 ubiquitylation has a direct role in promoting DSB repair events.

## Decoding DSB-induced histone ubiquitylation

**Figure 2:**
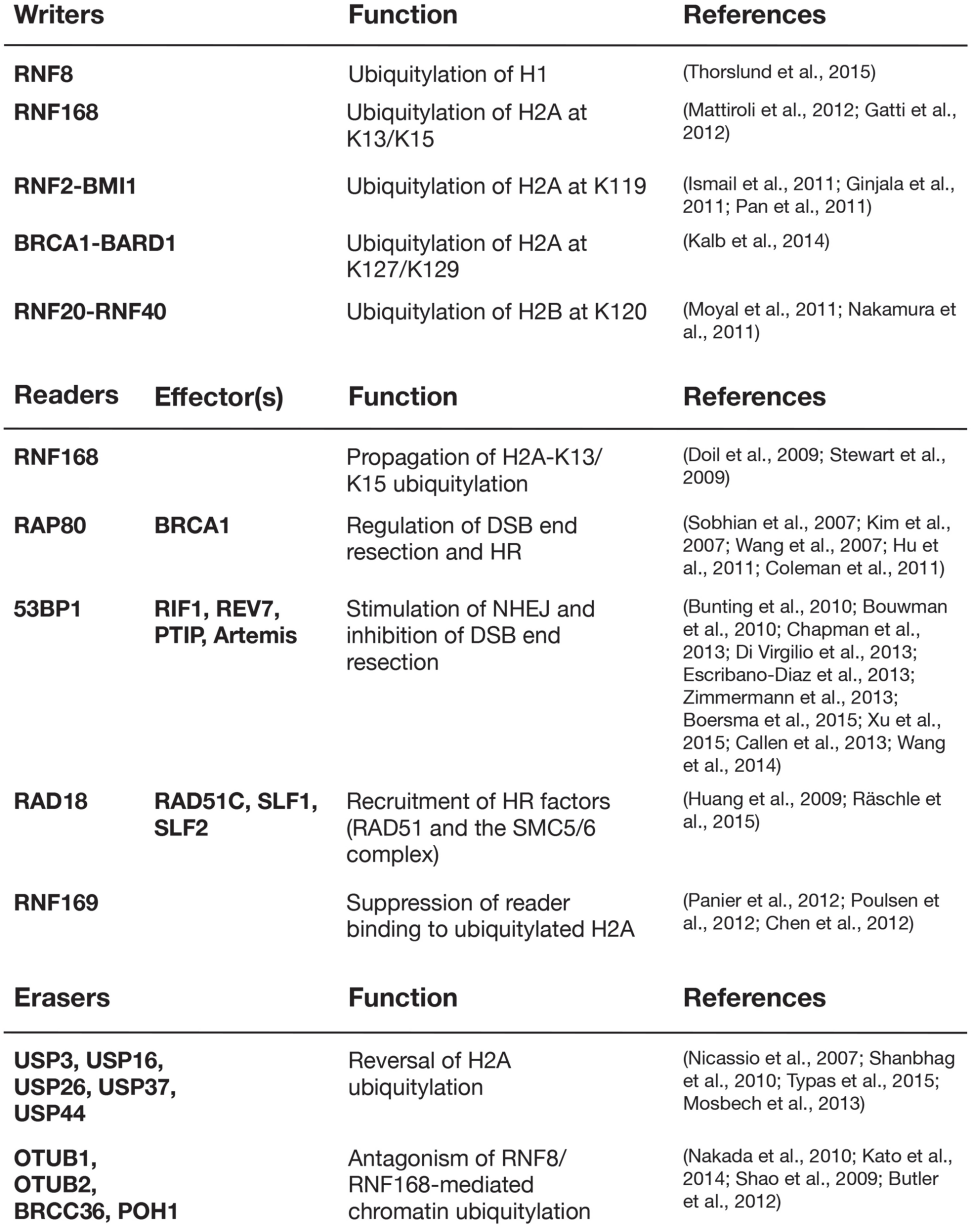
**Major writers, readers, and erasers of DSB-associated histone ubiquitylation**.

The ubiquitylation products of RNF8 and RNF168 provide affinity platforms at DNA damage sites for a range of factors that can be classified as “readers” of DSB-associated histone ubiquitylation marks (Figure 2). These reader proteins, several of whose functions are now relatively well understood, recognize DSB-induced chromatin ubiquitylation marks via intrinsic ubiquitin-binding domains (UBD) of the UIM-, MIU,- and UBZ-types (Dikic et al., 2009). Using the DSB signaling response as a model system for studying the specificity of ubiquitin recognition, it has been shown that target-specific binding to ubiquitylated ligands often relies on a dual interaction mode, in which the combination of low-affinity ubiquitin recognition by a UBD and target-binding specificity imparted by an adjacent module together enables specific, high-affinity interaction with damaged chromatin areas. Thus, several readers of RNF8/RNF168-dependent chromatin ubiquitylation in the DSB response were shown to contain so-called LR motifs that interact weakly with H2A or other factors, juxtaposed to their UBDs (Panier et al., 2012). In spatio-temporal order of recruitment, the range of chromatin ubiquitylation readers containing LR motifs in combination with UBDs is headed by RNF168, which binds K63-linked ubiquitin chains on histone H1 via its UDM1 domain (Thorslund et al., 2015). The subsequent RNF168-generated ubiquitylation products at DSB sites are then recognized by several factors, including RNF168 itself, RAP80, 53BP1, RAD18, and RNF169 (Panier et al., 2012; Fradet-Turcotte et al., 2013; Figure 2). The ability of RNF168 to both catalyze and recognize ubiquitin-modified H2A, the latter via its C-terminal UDM2 motif, may allow robust and efficient propagation of the DSB-induced H2A-ubiquitin signal along the chromatin fiber.

Several readers of RNF168-catalyzed ubiquitylation function as scaffolds for the assembly of DNA repair factor complexes at DSB sites. RAP80 recruits the key HR factor BRCA1 and other components of the multimeric BRCA1-A complex to damaged chromatin (Kim et al., 2007; Sobhian et al., 2007; Wang et al., 2007; Yan et al., 2007). RAP80 contains two tandem UIMs with a characteristic spacing that allows for specific binding to K63-linked ubiquitin chains *in vitro* (Kim et al., 2007; Sobhian et al., 2007; Sato et al., 2009; Sims and Cohen, 2009). Whether such linkage-specific chain recognition by RAP80 occurs *in vivo* is not fully clear, but this feature of RAP80 underscores the need to dissect the precise nature of the RNF168-catalyzed ubiquitylations on H2A and perhaps additional chromatin-bound proteins. Given that other readers of these modifications only rely on single UBDs, it seems unlikely that binding to K63-linked chains on H2A is a prerequisite for their recruitment to DSB sites. Indeed, it was shown that both RNF168, RAP80, and RAD18 can bind to RNF168-generated K27-linked ubiquitin chains on H2AK13/15 (Gatti et al., 2015).

53BP1, a key factor in DSB repair, does not contain a classical UBD, and its specific recruitment to RNF8- and RNF168-generated ubiquitin products at DSBs therefore long remained enigmatic. 53BP1 uses its tandem Tudor domain to bind mono- and di-methylated H4K20 (Botuyan et al., 2006), generated by SET8 (PR-SET7) and SUV4-20 h1/h2, respectively (Nishioka et al., 2002; Schotta et al., 2004; Yang et al., 2008). Since more than 80% of histone H4K20 is constitutively dimethylated in human cells (Yang et al., 2008), a range of potential mechanisms that would make H4K20me2 available for 53PB1 binding at damaged chromatin have been proposed. On the one hand, it has been suggested that SET8 and MMSET, another histone methyltransferase, are recruited to DSB sites where they methylate H4K20 *de novo* (Oda et al., 2010; Pei et al., 2011; Dulev et al., 2014; Tuzon et al., 2014). On the other hand, SET8, SUV4-20 h1/h2, and MMSET were all found to be dispensable for the DSB recruitment of 53BP1 in MEFs (Hartlerode et al., 2012). Other studies found that H4K20me2 is exposed at DSB sites through deacetylation of H4K16 (Hsiao and Mizzen, 2013) or by the displacement or degradation of the H4K20me2-binding factors L3MBTL1 and JMJD2A/B (Acs et al., 2011; Mallette et al., 2012). While none of these models are mutually exclusive, the precise scope of regulatory mechanisms impinging on H4K20 methylation at DSB sites and their relative importance for controlling 53BP1 recruitment were unclear. However, more recent findings established that 53BP1 not only relies on interaction with methylated H4K20, but also has an adjacent, cryptic ubiquitin recognition module (termed the UDR motif) that is required for its recruitment to sites of DNA damage (Fradet-Turcotte et al., 2013). The UDR preferentially recognizes H2AK15 ubiquitylation, which is specifically generated by RNF168 (Fradet-Turcotte et al., 2013). Several additional mechanisms contributing to 53BP1 accrual have been suggested, including direct SUMOylation and ubiquitylation of 53BP1 by PIAS4 and RNF168, respectively (Galanty et al., 2009; Bohgaki et al., 2013).

Other readers of histone ubiquitylation in the DSB response include the E3 ubiquitin ligases RAD18 and RNF169, both of which modulate the DSB response through mechanisms that do not appear to involve their catalytic activities. RAD18 promotes HR-mediated DSB repair through interaction with the RAD51 paralog RAD51C (Huang et al., 2009). More recently, RAD18 has been shown to be instrumental for recruiting the SMC5/6 cohesion complex to DNA damage sites downstream of RNF8/RNF168-generated histone ubiquitylation. This function requires two adaptor proteins, SLF1 and SLF2, which form a complex and physically bridge RAD18 and the SMC5/6 complex (Raschle et al., 2015). This recruitment pathway may promote faithful DSB repair and genome stability maintenance by suppressing illegitimate recombination events. Importantly, the involvement of RAD18 in DSB repair is distinct from its role in DNA damage bypass during DNA replication, where it facilitates exchanges of replicative DNA polymerases with UBD-containing damage-tolerant translesion DNA synthesis (TLS) polymerases such as DNA polymerase η (polη) at the replication fork by catalyzing PCNA monoubiquitylation in conjunction with RAD6 (Mailand et al., 2013). RNF169, a paralog of RNF168, accumulates at DSB sites by directly recognizing RNF168-generated ubiquitylation products, yet it does not cooperate with RNF168 in propagation of the H2A-ubiquitin mark (Chen et al., 2012; Panier et al., 2012; Poulsen et al., 2012). Instead, RNF169 appears to act as a negative regulator of downstream protein recruitment to DSBs by competing with other readers for binding to RNF168-generated ubiquitylation products, possibly helping to restrain the magnitude and propagation of the RNF8/RNF168-dependent DSB response.

## Erasers and regulators of DSB-induced histone ubiquitylation

Similar to the remarkable complexity by which RNF8 and RNF168 ubiquitylation products are laid down and interpreted, the regulation, maintenance, and removal of these modifications are governed by a range of different mechanisms. Several PTMs, including phosphorylation, SUMOylation, acetylation, and methylation are employed in the regulatory control of factors that affect the kinetics of DSB-associated chromatin ubiquitylation (Lukas et al., 2011; Polo and Jackson, 2011). For example, inactivating phosphorylations on RNF8 and 53BP1 by mitotic kinases suppress DSB-induced signaling and repair during mitosis. This has a critical role in protecting the genome from the formation of toxic repair products such as sister telomere fusions that can be otherwise generated during this window of the cell cycle (Orthwein et al., 2014).

The unique ability of RNF168 to both generate and interact with ubiquitylated H2A (Panier et al., 2012) allows for the dynamic and efficient spreading of DSB-induced chromatin marks along the chromatin fiber. At the same time, however, this begs the existence of regulatory mechanisms for keeping this potentially deleterious activity in check. One means of limiting the magnitude of the RNF8/RNF168-mediated chromatin ubiquitylation response involves the ubiquitin ligases UBR5 and TRIP12, which curb RNF168 expression by promoting its proteasomal degradation. In the absence of these factors, the DSB-specific histone ubiquitylation marks can undergo excessive spreading to entire damaged chromosomes as well as to adjacent ones (Gudjonsson et al., 2012). Interestingly, the destabilization of RNF168 by knockdown of HERC2 (Bekker-Jensen et al., 2010) is abrogated by co-depletion of TRIP12, suggesting that TRIP12 may target RNF168 molecules that are not in complex with HERC2 (Gudjonsson et al., 2012). In contrast, the deubiquitylating enzyme (DUB) USP34 has been speculated to stabilize RNF168 at DSB sites by counteracting proteasomal degradation of RNF168 through removal of K48-linked ubiquitin chains (Sy et al., 2013).

Several DUBs, including USP3, USP16, USP26, USP37, and USP44, target H2A for deubiquitylation in the context of the DDR, and when overexpressed, each of these DUBs efficiently reverse ubiquitin-dependent protein assembly at DSB sites (Figure 2). USP3, the first among these DUBs to be implicated in the DSB response, deubiquitylates H2A on a genome-wide scale (Nicassio et al., 2007; Doil et al., 2009; Lancini et al., 2014). Its ablation leads to increased DNA breakage, but to which extent this is due to hyperactivation of the DDR by ubiquitylated H2A or enhanced replication stress remains to be resolved (Nicassio et al., 2007; Lancini et al., 2014). USP16, which is predominantly cytoplasmic during interphase, has been implicated in deubiquitylation of H2A during mitosis, but has also been shown to counteract transcriptional silencing associated with DSB-induced histone ubiquitylation (Cai et al., 1999; Joo et al., 2007; Shanbhag et al., 2010). In addition, it was suggested that USP16 interacts with and is regulated by HERC2 and acts downstream of RNF8 and RNF168 at DSB sites (Zhang et al., 2014). USP44 was identified in a screen for DUBs whose overexpression prevent 53BP1 recruitment to DSB sites and has been shown to reverse ubiquitylation of both H2A and H2B (Fuchs et al., 2012; Mosbech et al., 2013). The OTU family DUB OTUB1 is highly active against K48-linked ubiquitin chains but negatively impacts the DSB response in a non-canonical manner independent of its catalytic activity. OTUB1 associates with and inhibits a range of E2s, including Ubc13 and UbcH5, thereby preventing RNF168 recruitment to, and H2A ubiquitylation at, DSBs (Nakada et al., 2010; Juang et al., 2012; Sato et al., 2012; Wiener et al., 2012). OTUB2 and BRCC36, a component of the BRCA1-A complex, both preferentially cleave K63-linked ubiquitin chains and have been proposed to antagonize RNF8-Ubc13-dependent ubiquitylation at DSB sites (Shao et al., 2009; Kato et al., 2014). Thus, an elaborate array of DUBs are engaged in regulating and fine-tuning the RNF8/RNF168-mediated chromatin ubiquitylation response to DSBs, emphasizing its biological importance and plasticity. The collective antagonistic actions of these DUBs may both allow cells to set a basal threshold for triggering the full-blown ubiquitin-dependent response to DSBs, and to regulate and fine-tune its magnitude during the course of lesion repair.

## Histone ubiquitylation and DSB repair pathway choice

By mediating the recruitment of a range of key DSB repair factors, including BRCA1, 53BP1 and associated proteins, chromatin ubiquitylation at DNA damage sites by RNF8 and RNF168 plays an important role in promoting DSB repair pathway efficiency and utilization. The choice between NHEJ and HR for the repair of a DSB is primarily made at the level of DSB end protection, which favors NHEJ, and end resection, which commits the break to repair by HR (Chapman et al., 2012b). 53BP1, whose relocalization to DSB sites is strongly dependent on RNF8/RNF168-mediated chromatin ubiquitylation, functions at the crossroads of DSB pathway choice. Accordingly, 53BP1 actively promotes NHEJ in several contexts while inhibiting BRCA1-mediated HR through mechanisms that are still being elucidated. 53BP1 limits HR by posing a barrier for the DSB end resection machinery to approach the break sites, a function that is specifically antagonized by BRCA1 under HR-permissive conditions during the S and G2 phases, causing the relocalization of 53BP1 to the periphery of IRIF (Cao et al., 2009; Bouwman et al., 2010; Bunting et al., 2010; Chapman et al., 2012a; Karanam et al., 2012). Several effector proteins collaborate with 53BP1 in these NHEJ-promoting processes. The proteins RIF1 and PTIP bind 53BP1 via non-overlapping, phosphorylation-dependent interactions involving a large cluster of ATM phosphorylation sites in its N-terminal half (Munoz et al., 2007; Callen et al., 2013; Chapman et al., 2013; Di Virgilio et al., 2013; Escribano-Díaz et al., 2013; Feng et al., 2013). While RIF1 appears to act as an HR inhibitor that antagonizes BRCA1, PTIP mediates pro-NHEJ functions of 53BP1 involving its binding to the nuclease Artemis, a known NHEJ component (Wang et al., 2014). PTIP is responsible for mutagenic DNA repair processes such as the fusion of unprotected telomeres during mitosis (Callen et al., 2013). On the other hand, the requirement of 53BP1 for productive CSR in developing B cells, a process that impinges on the repair of a programmed DSB by NHEJ, appears to be channeled mainly through RIF1 (Callen et al., 2013; Chapman et al., 2013; Di Virgilio et al., 2013; Escribano-Díaz et al., 2013). Recently, REV7 (also known as MAD2L2) was found to act downstream of 53BP1 and RIF1 in inhibiting end resection and promoting CSR, independent of its role in TLS in conjunction with REV3L (Boersma et al., 2015; Xu et al., 2015). However, because REV7 does not appear to form complexes with 53BP1 and RIF1, the precise mechanism by which it promotes these processes remains to be established. The emerging linear RNF8-RNF168-53BP1-RIF1-REV7 cascade also mediates the DDR at uncapped telomeres, promoting illegitimate NHEJ-dependent fusions of deprotected chromosome ends that lead to massive genome instability (Peuscher and Jacobs, 2011; Okamoto et al., 2013; Zimmermann et al., 2013; Boersma et al., 2015).

BRCA1 binds to resected DNA independently of the RAP80-containing BRCA1-A complex, promoting HR in complex with either CtIP or BACH1 (Huen et al., 2010). RAP80 prevents hyper-recombination by limiting the access of BRCA1 to end-resected DSBs (Coleman and Greenberg, 2011; Hu et al., 2011). When bound to its partner protein BARD1, BRCA1 is active as an E3 ubiquitin ligase (Wu et al., 1996), and cancer-predisposing germline mutations in the *BRCA1* gene cluster in its N-terminal RING domain that underlies this activity (Maxwell and Domchek, 2012). However, ES cells expressing an E3 ligase-deficient BRCA1 allele containing an I26A mutation in the RING domain, which preserves the binding to BARD1, are viable, unlike *BRCA1* null cells (Reid et al., 2008). Mice expressing this BRCA1 mutant display no increase in tumor formation, indicating that the ubiquitin ligase activity of BRCA1 may not be absolutely essential for its tumor suppressor function (Shakya et al., 2011). In addition, the nature of the physiologically relevant BRCA1 E3 ligase substrate(s) remains unclear. Recently, BRCA1 was found to ubiquitylate H2A *in vitro* at the C-terminal residues K127 and K129 (Kalb et al., 2014), sites that are not known to be targeted by other H2A ubiquitin ligases. In line with this finding, it has been known for some time that expression of a chimeric protein in which ubiquitin is fused in-frame to the C-terminus of H2A rescues the defect in heterochromatin silencing observed in cells lacking BRCA1 (Zhu et al., 2011). Whether BRCA1-mediated H2A K127/K129 ubiquitylation has any functional role in DSB repair is therefore an important question that awaits clarification.

As RNF168 is required for the recruitment of both 53BP1 and the BRCA1-A complex to DSBs, it is feasible that this protein also plays a role in the regulation of DSB repair pathway choice. Interestingly, RNF168 depletion in BRCA1-deficient cells rescued HR and RAD51 IRIF formation, similar to 53BP1 loss, but was not able to restore HR in cells lacking CtIP, RAD50, RAD51, or BRCA2 (Muñoz et al., 2012). A truncated form of RNF168 unable to form IRIF still promoted HR, suggesting that the function of RNF168 in HR does not require its localization to DSB sites (Muñoz et al., 2014). Moreover, ectopic expression of RNF168 or 53BP1 in BRCA1-deficient cells exacerbates their hypersensitivity to PARP inhibitors by promoting 53BP1 accrual and suppression of resection (Zong et al., 2015). Although some of the DUBs that antagonize H2A ubiquitylation and/or RNF168 recruitment to DSB sites likely play a role in regulating the balance between 53BP1 and BRCA1 and DSB repair pathway choice, only a few have been directly implicated in this process. POH1 (also known as PSMD14) is a proteasome-associated DUB that accumulates at DSB sites, and whose knockdown gives rise to enlarged 53BP1 foci, suggesting that the proteasome is involved in the turnover of ubiquitin conjugates at sites of DNA damage (Butler et al., 2012; Galanty et al., 2012). POH1 collaborates with BRCA1 to overcome the 53BP1-mediated barrier to DNA end resection, leading to DSB repair by HR (Kakarougkas et al., 2013). USP26 and USP37 also accumulate at DSBs and deubiquitylate H2A. These DUBs have been suggested to counteract RNF168-dependent BRCA1 repression by RAP80, thereby promoting HR via BRCA1-dependent loading of PALB2-BRCA2 and RAD51 onto the resected DNA (Typas et al., 2015). While tremendous progress has thus been made toward understanding the mechanisms underlying DSB repair pathway choice that are critically dependent on the tug of war between BRCA1 and 53BP1, many questions remain about how they exert their functions in this process. In particular, despite its clinical importance as a tumor suppressor that has been recognized for decades, the reason why BRCA1 is so important for aborting tumorigenic events remains enigmatic. Further efforts to decipher the mechanisms underlying the antagonistic relationship between 53BP1 and BRCA1 in DSB signaling should help to clarify the role of BRCA1 in protecting against breast and ovarian cancers, and may lead to improved cancer treatment strategies.

In addition to histone ubiquitylation mediated by RNF8, RNF168, and other E3 ubiquitin ligases, the choice between NHEJ and HR for repair of individual DSBs is also controlled and regulated by a range of other ubiquitin-dependent processes (reviewed in detail in Schwertman et al., 2016). For example, ubiquitin has a direct key role in suppressing HR during G1 phase. This is mediated by KEAP1, a Cullin 3-RING ubiquitin ligase (CRL3) complex substrate adaptor (Cullinan et al., 2004; Kobayashi et al., 2004; Zhang et al., 2004), which ubiquitylates PALB2 to inhibit the interaction between BRCA1 and PALB2-BRCA2 (Orthwein et al., 2015). USP11, a DUB that has previously been implicated in HR and interacts with BRCA2 (Schoenfeld et al., 2004; Wiltshire et al., 2010), deubiquitylates PALB2 but is degraded in G1 upon DSB formation, thereby effectively suppressing HR specifically during this window of the cell cycle (Orthwein et al., 2015). Likewise, the E3 ligase RNF138 was recently shown to play an important role in promoting DSB end resection, through its dual ability to ubiquitylate Ku80, promoting displacement of the Ku70-Ku80 complex from DSB ends, and the resection factor CtIP, enabling its recruitment to DSB sites (Ismail et al., 2015; Schmidt et al., 2015). The integration of multiple ubiquitin-dependent signaling processes targeting chromatin and DNA repair factors thus provides a critical regulatory framework for controlling DSB repair pathway utilization and efficiency during the cell cycle.

## Future perspectives

Histone ubiquitylation by the RNF8/RNF168 cascade forms the nexus of a multipronged signaling pathway that entails the recruitment of many important repair factors to DNA damage sites, playing a central role in regulating the choice and efficiency of DSB repair mechanisms. A decade of studies of the RNF8/RNF168 pathway and its role in shaping the dynamic chromatin ubiquitylation landscape in the vicinity of DSBs has revealed many important insights into its complex spatio-temporal organization and regulation, as well as its impact on genome stability maintenance. However, many questions about the workings of this signaling response remain. Although it is becoming increasingly clear that RNF8 and RNF168 target different histones at DSB-surrounding chromatin areas, it is possible that they may have additional substrates relevant to their DSB signaling and repair functions. In addition, while RNF168 specifically ubiquitylates N-terminal lysines in H2A-type histones, the residue(s) in linker histones targeted by RNF8 are not yet known. For H2A, however, the nature of the physiologically relevant ubiquitin modifications assembled by RNF168 needs to be further addressed, with both H2A monoubiquitylation and polyubiquitylation via K63- and K27-linkages potentially playing important roles in the context of DSB responses. Whether other DSB-responsive ubiquitin ligases, including RAD18, HERC2, and RNF169, are actively engaged in ubiquitylating histones or other chromatin-bound factors at DSB sites also awaits clarification. Finally, it remains to be conclusively established whether some readers of RNF168-catalyzed ubiquitylation products at DSB sites, including RAP80 and RAD18, mainly recognize ubiquitylated H2A or perhaps other RNF168-modified proteins on chromatin. It is also possible that there are as-yet unknown readers of RNF8- and RNF168-dependent ubiquitylation marks at damaged chromatin. Structural studies will be of key value for a better understanding of the relationships between ubiquitylated histones and their writers, readers and erasers in DSB repair. The recent breakthroughs in cryo-EM technology hold promise that it will be feasible to obtain structures of ubiquitin-modified histones and nucleosomes in complex with such proteins and complexes, thus paving the way for atomic-level resolution of ubiquitin-dependent DSB signaling responses.

The identification of H1-type linker histones as targets of RNF8-dependent K63 polyubiquitylation adds a new and dynamic dimension to the histone code for DSB repair. While the ubiquitylated forms of H1 histones can serve as an anchor for initial RNF168 recruitment to the break sites, it is conceivable that DSB-induced linker histone modifications may also play a role in remodeling chromatin structure to render it more permissive for efficient repair. To this end, it has been shown that murine cells lacking three of the six linker histone isoforms are more resistant to DNA damage, and that the K63-ubiquitylated forms of H1 are more loosely associated with chromatin than their unmodified counterparts (Murga et al., 2007; Thorslund et al., 2015). Thus, dynamic DNA damage-induced modifications of linker histones by ubiquitin and perhaps other PTMs, numerous of which have been mapped by proteomic studies (Harshman et al., 2013), might promote restructuring of chromatin composition and compaction to facilitate the accessibility of the DSB repair machinery. How linker histones and their modifications impact chromatin structure dynamics during the course of DNA repair is an underexplored issue that merits further attention in the coming years.

Downstream of RNF8/RNF168-dependent chromatin ubiquitylation, the molecular transactions governing DSB repair pathway choice as a function of the well-established tug of war between 53BP1 and BRCA1 and their associated proteins are an intensely studied and clinically relevant topic, with many important questions still to be addressed. In particular, the mechanism(s) by which BRCA1 promotes DSB end resection, HR and tumor suppression remain incompletely understood. From the perspective of chromatin ubiquitylation, an especially pertinent issue that has so far eluded clear-cut answers concerns the role and substrate(s) of BRCA1-BARD1 E3 ligase activity in DSB repair. BRCA1 has been shown to contribute to chromatin ubiquitylation at DSB sites and modifies C-terminal lysine residues in H2A (Morris and Solomon, 2004; Polanowska et al., 2006; Kalb et al., 2014). Resolving if and how such BRCA1-mediated ubiquitylation at damaged chromatin contributes to its role in DSB repair may provide much-needed insights into the molecular mechanisms underlying its pro-HR function.

As many key players involved in the ubiquitin-dependent chromatin response to DSBs have now been identified and characterized, a major goal for the coming years will be to further delineate the mechanisms underlying these regulatory signaling processes and their integration into biologically meaningful cellular responses and circuitries that enable genome stability protection after DSBs in a ubiquitin-driven manner.

## Author contributions

All authors listed have made substantial, direct and intellectual contribution to the work, and approved it for publication.

### Conflict of interest statement

The authors declare that the research was conducted in the absence of any commercial or financial relationships that could be construed as a potential conflict of interest.
